# Distinct Microbial Assemblage Structure and Archaeal Diversity in Sediments of Arctic Thermokarst Lakes Differing in Methane Sources

**DOI:** 10.3389/fmicb.2018.01192

**Published:** 2018-06-07

**Authors:** Paula B. Matheus Carnevali, Craig W. Herbold, Kevin P. Hand, John C. Priscu, Alison E. Murray

**Affiliations:** ^1^Division of Earth and Ecosystem Sciences, Desert Research Institute, Reno, NV, United States; ^2^Department of Microbiology and Ecosystem Science, University of Vienna, Vienna, Austria; ^3^Jet Propulsion Laboratory, California Institute of Technology, Pasadena, CA, United States; ^4^Department of Land Resources and Environmental Sciences, Montana State University, Bozeman, MT, United States

**Keywords:** methane, thermokarst, lake, sediments, Arctic, Alaska, archaea, diversity

## Abstract

Developing a microbial ecological understanding of Arctic thermokarst lake sediments in a geochemical context is an essential first step toward comprehending the contributions of these systems to greenhouse gas emissions, and understanding how they may shift as a result of long term changes in climate. In light of this, we set out to study microbial diversity and structure in sediments from four shallow thermokarst lakes in the Arctic Coastal Plain of Alaska. Sediments from one of these lakes (Sukok) emit methane (CH_4_) of thermogenic origin, as expected for an area with natural gas reserves. However, sediments from a lake 10 km to the North West (Siqlukaq) produce CH_4_ of biogenic origin. Sukok and Siqlukaq were chosen among the four lakes surveyed to test the hypothesis that active CH_4_-producing organisms (methanogens) would reflect the distribution of CH_4_ gas levels in the sediments. We first examined the structure of the little known microbial community inhabiting the thaw bulb of arctic thermokarst lakes near Barrow, AK. Molecular approaches (PCR-DGGE and iTag sequencing) targeting the SSU rRNA gene and rRNA molecule were used to profile diversity, assemblage structure, and identify potentially active members of the microbial assemblages. Overall, the potentially active (rRNA dominant) fraction included taxa that have also been detected in other permafrost environments (e.g., Bacteroidetes, Actinobacteria, Nitrospirae, Chloroflexi, and others). In addition, Siqlukaq sediments were unique compared to the other sites, in that they harbored CH_4_-cycling organisms (i.e., methanogenic Archaea and methanotrophic Bacteria), as well as bacteria potentially involved in N cycling (e.g., Nitrospirae) whereas Sukok sediments were dominated by taxa typically involved in photosynthesis and biogeochemical sulfur (S) transformations. This study revealed a high degree of archaeal phylogenetic diversity in addition to CH_4_-producing archaea, which spanned nearly the phylogenetic extent of currently recognized Archaea phyla (e.g., Euryarchaeota, Bathyarchaeota, Thaumarchaeota, Woesearchaeota, Pacearchaeota, and others). Together these results shed light on expansive bacterial and archaeal diversity in Arctic thermokarst lakes and suggest important differences in biogeochemical potential in contrasting Arctic thermokarst lake sediment ecosystems.

## Introduction

The thawing front (talik or thaw bulb) of permafrost underneath shallow (~2–3 m deep) arctic thermokarst lakes is an active area of carbon (C) cycling, where carbon dioxide (CO_2_) and methane (CH_4_) are produced and consumed by microbially driven processes (Bretz and Whalen, 2014; Lofton et al., 2014; Heslop et al., 2015). Knowledge of the organisms and communities driving these processes has only recently begun to reveal the extent of microbial communities diversity and structure in thermokarst lakes or similar saturated permafrost features. Initial findings suggest that CH_4_ cycling microorganisms are important components of these systems. For example, type I and type II bacterial methanotrophs were found in the sediments of an Alaskan Arctic thermokarst lake (Qalluuraq Lake) that emits ebullient CH_4_ (He et al., 2012b). In another study, CH_4_ producers (methanogens) affiliated with Methanomicrobiales and Methanosarcinales were observed in sediments of small, shallow ponds in the Canadian Arctic (Negandhi et al., 2013). Likewise, abundant methanogen SSU rRNA gene sequences and high methanogenesis transcript to gene ratios were detected in thermokarst bog soils in the Tanana Flats in central Alaska (Hultman et al., 2015). Denitrifiers and sulfate (${\text{SO}}_{4}^{2-}$) reducers co-occurred in the bog soils, demonstrating the interconnectedness between biogeochemical processes in the soils.

The Coastal Plain of northern Alaska is a large (i.e., 22,000 square kilometers; Wang et al., 2012) matrix of permafrost peppered with thermokarst lakes, some of which produce either ebullient CH_4_ generated by present-day biogenic processes (e.g., Siqlukaq Lake) or as a result of release by thermogenic processes (e.g., Sukok Lake, which overlies a portion of the national petroleum reserve; Matheus Carnevali et al., 2015). To date, permafrost features in this area rather than thermokarst lakes have received the most attention, given the need for gauging response of permafrost to warming temperatures (Graham et al., 2012; Lipson et al., 2013).

As part of an interdisciplinary study on CH_4_-related process in this region, we hypothesized that the microbial assemblages in the sediments of the ebullient thermogenic CH_4_ lakes would be distinct from the biogenic CH_4_ producing lakes. To address this, we first assessed archaeal diversity across four arctic thermokarst lakes (three dominated with biogenic CH_4_ sources and one with a thermogenic source; Matheus Carnevali, 2015; Matheus Carnevali et al., 2015; Elvert et al., 2016; Hand, unpublished results), and then based on those results, we targeted our focus to two lakes, Siqlukaq (Siq) and Sukok (Suk). In addition to different CH_4_ sources, sediments of these two lakes harbor contrasting levels of TOC (between 0.2 and 15 wt. %) (Matheus Carnevali et al., 2015). Our analyses targeted both ribosomal RNA genes and the ribosomal RNA molecule of both bacteria and archaea, provided that DNA from non-viable microbial cells may be present in the environment (Willerslev et al., 2004; Blazewicz et al., 2013). In comparison to mRNA, the more environmentally stable rRNA where detected in appreciable levels, may be indicative of potentially active bacteria and archaea (Juottonen et al., 2008; Stoeva et al., 2014; Crevecoeur et al., 2016). For instance, taxonomic affiliations of SSU rRNA iTags and the mRNA fraction (metatranscriptome) assigned to taxonomic bins have been observed to correspond (Tveit et al., 2012; Hultman et al., 2015). We also took a deeper look into the phylogenetic diversity of the Archaea by sequencing cloned archaeal rRNA genes (partially covered).

## Materials and methods

### Sampling sites and sediment core collection

Shallow (~2–3 m deep) arctic thermokarst lakes on the north slope of Alaska were sampled during three different field campaigns (April and October 2010, and October 2011; Table 1). Three of the lakes sampled (Ikroavik, Sukok, and Siqlukaq) are located southeast of Barrow, and the fourth lake (Qalluuraq) is located further south near Atqasuk. Sediment cores were collected through holes drilled in the frozen surface lake ice (0.68–1.40 m in April 2010, 0.25 m in October 2010, and 0.08–0.15 m in October 2011) using a universal percussion corer (Aquatic Research Instruments). Cores were transported by snow mobile to the Naval Arctic Research Laboratory (NARL) and stored frozen at −80°C until subsampling. In depth analyses here focused on sediment cores collected in October 2011: one site in Siqlukaq (Siq) and two sites in Sukok, a site nearby an open CH_4_ seep (SukS) and a site ~1 km southwest from the seep (SukB). Sukok is underlain by the Walakpa natural gas field and displays ebullition throughout the year, especially in the SukS area. Matheus Carnevali et al. (2015) conducted the first studies on CH_4_ source from these lakes. The coring sites, sediment intervals sampled, and associated downstream analyses are listed in Table 1.

**Table 1:** Sediment coring sites, core depths sampled, and associated molecular analyses conducted.

**Date sampled**	**Site**	**Estimated depth sub-sampled (cm)^a^**	**Sample ID**	**DNA**	**RNA**	**aRNA**	**iTag SSU rRNA V4 region (505F-806R)**	**Clone libraries Archaea SSU rRNA gene (109F-915R)^b^**	**Clone libraries Archaea SSU rRNA gene (20F-958R)^b^**	**DGGE Archaea SSU rRNA V3 region (GC347F-UNIV519R)^c^**
Apr-2010	Ikroavik	1–17	Ikr10-A	+	–	–	–	+^ii^	–	+
Apr-2010	Qalluuraq	1–58	Qal10	+	–	–	–	–	–	+
Apr-2010	Sukok C	1–31	SukC10	+	–	–	–	–	–	+
Oct-2010	Ikroavik	2–28	Ikr10-O	+	–	–	–	+^ii^	–	+
Oct-2010	Siqlukaq	10–50	Siq10	+	–	–	–	+^ii^	–	+
Oct-2010	Sukok Seep	2–106	SukS10	+	–	–	–	+^ii^	–	+
22-Oct-2011	Siqlukaq	6–7	Siq-U	+	+	+	+^i^	–	+^i^	–
	Siqlukaq	13–14	Siq-M	+	+	+	+^i^	–	+^i^	–
	Siqlukaq	23–24	Siq-L	+	+	+	+^i^	–	+^i^	–
24-Oct-2011	Sukok B	6–8	SukB-U	+	+	+	+^i^	–	+^i^	–
	Sukok B	11–14	SukB-M	+	+	+	+^i^	–	+^i^	–
	Sukok B	21–24	SukB-L	+	+	+	+^i^	–	+^i^	–
20-Oct-2011	Sukok Seep	7–9	SukS-U	+	–	+	+^i^	–	+^i^	–
	Sukok Seep	16–19	SukS-M	+	–	+	+^i^	–	+^i^	–
	Sukok Seep	22–24	SukS-L	+	–	+	+^i^	–	+^i^	–

*The geographic positions of the 2011 coring sites were reported in (Matheus Carnevali et al., 2015). 2010 coring sites were: Ikr10: 71° 14.036′N, 156° 38.349′W, Siq10: 71° 10.486′N, 156° 53.891′W, SukS10: 71° 04.455′N, 156° 49.250′W, and Qal10: 70° 22.672′N, 157° 20.927′W (60 miles to the south). The shaded area highlights samples used for iTag surveys*.

a *Two to 10 subsamples were collected per core. See Materials and Methods section for details about the number of samples per depth interval*.

b *Samples from discrete depth intervals were pooled for clone libraries construction to study Archaea phylogeny in arctic thermokarst lakes. Latitude and longitude of the coring sites were reported in Matheus Carnevali et al. (2015)*.

c *Discrete depth intervals were loaded onto the gel to survey archaeal diversity (see Figure S2 for details)*.

i *DNA Extraction method i*.

ii *DNA Extraction method ii*.

### Nucleic acid extractions

Over the course of this study two DNA extraction protocols and one RNA extraction protocol were executed. The different DNA extraction protocols were used for separate downstream purposes (indicated with numerals i and ii).

*DNA extraction (i):* DNA from 2011 sediments was used for Bacteria and Archaea SSU rRNA gene iTag sequencing and archaeal SSU rRNA gene clone libraries. We studied the upper sediment layers (0–30 cm) given observed differences in CH_4_ production in these layers (Matheus Carnevali et al., 2015). DNA was extracted from three sections (U-upper, M-middle, L-lower; ~1 g ea.) of the first 30 cm of each 2011 sediment core (Siq, SukS, and SukB; Table 1) using a Power Soil DNA isolation kit (MoBio). DNA was quantified (Quant-iT; Picogreen Assay Kit; Life Technologies) on a fluorescence reader (Spectromax Gemini, Molecular Devices). All comparisons between sites were made with DNA extracted using this method. Additional subsamples from the same core sections preserved at −80°C in sucrose lysis buffer (SLB, 40 mM EDTA, 50 mM Tris-HCl, 0.75 M sucrose) were used to generate DNA for archaeal rRNA gene clone libraries.

*DNA extraction (ii):* 2010 sediments were used for archaeal PCR-DGGE profiling and 2010 archaeal SSU rRNA gene clone libraries. Subsamples from 2010 cores (1 g ea.; Table 1) were collected from the top ~0–60 cm of Qal10 (6 samples; April), the top ~0–20 cm of Ikr-10A (3 samples; April), the top ~0–30 cm (2 samples; October) of Ikr10-O, the top ~0–30 cm of SukC (4 samples; April), the top ~10–50 cm of Siq10 (3 samples; October), and along the length (~0–110 cm) of the SukS10 core (10 samples; October). Genomic DNA was extracted using phenol-chloroform purification (Massana et al., 1997) and quantified (Nanodrop, ThermoFisher Scientific).

#### RNA extraction: SSU rRNA bacteria and archaea iTag sequencing

To target potentially active members of the microbial assemblage, total RNA was extracted from three sections (U-upper, M-middle, L-lower; 4 g ea.) of the first 30 cm of the 2011 cores (Siq, SukB, and SukS; Table 1). The RNA PowerSoil Total RNA Isolation Kit (MoBio) was used for nucleic acid extraction omitting the purification steps (Supplemental Methods), and included reagent-only extraction controls. Total nucleic acid extracts were treated 2–3 times with RNase-free DNase I (4 units each; Life Technologies) at 37°C in 100 μL reaction volumes for 30 min. Total RNA was purified from DNase enzyme following standard procedures (Sambrook et al., 1989) then quantified (Quant-iT RiboGreen RNA Assay Kit) on a fluorescence reader. Extraction controls were also subjected to DNAse treatment.

### Reverse transcription and RNA amplification of the SSU rRNA gene

To obtain cDNA from rRNA, total RNA was reverse transcribed with SuperScript III Reverse Transcriptase (Life Technologies) following manufacturer's instructions using a SSU rRNA-specific directed primer (806R; Caporaso et al., 2010). Siq11-U, -M, and -L samples were diluted 1/10 before the reverse transcription step to prevent enzymatic inhibition observed in previous experiments. cDNA products were verified on an agarose gel and quantified (Quant-iT PicoGreen dsDNA Assay Kit). Reagent controls were also subjected to reverse transcription and PCR in which subsequent amplification products were not detected.

RNA yields from five of the nine samples (Siq-U, Siq-M, SukB-U, SukB-M, and SukB-L) were sufficient to generate cDNA directly from the total RNA to be used in iTag sequencing. We then amplified the total RNA for direct comparison of the sequence-based identities of the potentially active bacteria and archaea across the set of 9 lake sediment samples. The MessageAmp II aRNA Amplification Kit (Life Technologies) was used according to manufacturer's instructions and final RNA products (aRNA) were quantified (Quant-iT RiboGreen RNA Assay Kit, Invitrogen) on a fluorescence plate reader. Amplified RNA was then reverse transcribed to cDNA with primer 806R (Caporaso et al., 2010).

DNA (9 samples) and cDNA from both RNA (5 samples) and aRNA (9 samples) were sent to the Joint Genome Institute (JGI, Walnut Creek, CA) for library preparation and paired-end (2 × 250 bp) MiSeq Illumina sequencing of the variable region 4 (V4) SSU rRNA using primers 515F (5′-GTGCCAGCMGCCGCGGTAA-3′) and 806R (5′-GGACTACHVGGGTWTCTAAT-3′) following Caporaso et al. (2010). Table 1 lists the samples sequenced. The iTag libraries were submitted to GenBank SRA under accession numbers SRR5313010-SRR5313032.

### iTag sequence processing and statistical analyses

Sequence processing included removal of PhiX contaminants and Illumina adapters, then quality was evaluated using the FastQC software (Babraham Bioinformatics). Trimmomatic (Bolger et al., 2014) was then used in the single end mode to filter the low quality sequences resulting in 3,834,911 (85.8%) out of 4,469,505 sequences that passed the quality-filtering step. Sequence file headers formatted with an in-house script were imported into Mothur (Kozich et al., 2013) where paired end reads were mated into contigs, chimeras were removed, sequences were initially classified into the Greengenes gg_13_8_99 taxonomy (Silva v. 123 was referenced for updated archaeal taxonomy), and sequences were clustered into operational taxonomic units (OTUs) at a distance of 0.03. These sequences were used to create an OTU database containing OTU occurrence, a representative sequence, and the consensus taxonomy (Kozich et al., 2013). The Bacterial taxonomy of OTUs that were identified as “potentially active” was revised later using Silva v. 132 taxonomy.

Read depths between different samples were normalized by bootstrap resampling (500 bootstraps) using R (R Core Development Team, 2015) for β-diversity analyses following the methods and custom scripts developed by Herbold et al. (2014). To reduce the effect of sequencing error, OTUs with 5 sequences or less among all of the samples (DNA, aRNA, and RNA from all sites) were removed prior to resampling, resulting in a total of 1,213,686 sequences among the 23 samples. The sample() command was then used to bootstrap each sample at 75% of the smallest library size using the observed OTU counts as a probability vector. To account for stochastic effects on the observance of rare OTUs, the total size of the sample (observed + hidden) was estimated using the Chao1 index (Chao, 1984). The non-observed proportion was evenly divided between all OTUs (observed and non-observed) so that non-observed OTUs in each sample possessed a non-zero probability of being resampled into a bootstrapped community.

Non-metric multidimensional scaling (NMDS) ordinations of DNA and aRNA sequences were generated using the Bray-Curtis dissimilarity values calculated with 20 re-samplings for each sample. Pairwise differences in Bray-Curtis dissimilarity between sites were tested with the Mann-Whitney test on resampled communities. Significance thresholds were defined by the Benjamini-Hochberg procedure with a false discovery rate of 0.05. These statistical analyses were conducted in R using the vegan package as well as the p.adjust(method = “fdr”) commands included in the base stats package.

### aRNA to DNA ratio as an indicator of potential activity

We used both aRNA and DNA sequence types to understand the variance in composition of potentially active organisms between lake sediment sites. To identify OTUs that may have been potentially active in these lake sediments communities were resampled 100,000 times, at 75% of the smallest library size among DNA and aRNA samples (resample size = 31,339 sequences). During each resampling, the resampled aRNA sequences were compared to resampled DNA sequences for each OTU. *P*-values reflecting the significance of aRNA sequences being greater than DNA sequences, and thus “activity” was estimated by counting the number of times out of 100,000 the resampled aRNA sequences were less than the resampled DNA sequences. The Benjamini–Hochberg procedure was used to determine a *p-*value equivalent to false discovery rate (FDR) = 0.05 (*p* ≤ 0.00019). Thus, if the aRNA values were less than the DNA values less than 19 times out of the 100,000 resamplings, that OTU in that sample was classified as “active.” OTUs that may have been overrepresented as a result of RNA amplification were identified by plotting the five samples for which both RNA and aRNA data sets were available (i.e., OTUs 20, 22, 31, 70, 89, and 170 in SukB; Figure S1). These OTUs were included in downstream analyses, but caution was applied in their interpretation (i.e., OTUs with aRNA sequences >100 and RNA sequences <10 were considered suspicious). Additionally, OTUs whose aRNA sequences were exceptionally high (>1,000; represented as 1,000 on **Figure 2**) than their DNA sequences (< 2), as well as OTUs with DNA sequences < 5, were not taken into consideration. OTUs with aRNA to DNA ratios >1 were broken into three groups as proxies to enable consistent comparisons of varying aRNA:DNA levels between sites. OTUs with ratios >2 and aRNA sequence occurrence levels over 500 were assigned to Group 1 (G1), those with ratios >2 and aRNA sequence levels between 250 and 500 were assigned to Group 2 (G2), and those with ratios between 1 and 2 with aRNA sequence levels ≥250 and DNA levels ≥250 were assigned to Group 3 (G3). OTUs with aRNA levels ≥1,000 or DNA levels ≤5 were not assigned a G-level given the potential for aRNA over-amplification and RNA to DNA ratio inflation with low DNA signals.

### PCR-DGGE of archaeal SSU rRNA genes

To survey the structure of the archaeal assemblages in the thermokarst lake sediments, core sections collected from all lakes sampled in 2010 (Table 1) were analyzed by PCR-DGGE analysis of the V3 region of the archaeal SSU rRNA gene. Details are presented in the supplemental methods, though briefly, sample loads were normalized to 600 ng of DNA per sample and run a 8% polyacrylamide, 30–65% denaturing gradient gel for 16 h at 60 volts, then the SYBR-Gold-stained gel was visualized and analyzed using GelCompar II software (Applied Maths).

### Archaeal SSU rRNA gene clone library construction and phylogenetic analyses

Archaeal clone libraries were prepared for each of the 2010 and 2011 sets of sediment cores (Table 1) in order to maximize the scope of the phylogenetic investigation. Briefly, sediment DNA extracts of the 2010 and 2011 sediment intervals (5 μL each) were pooled for PCR and non-directional cloning (see Supplemental Methods section for additional details). Sequencing was unidirectional and resulted in sequences that partially overlapped. To obtain a broad coverage of Archaea phyla, the 2010 library was generated using SSU rRNA gene primers 109F (Großkopf et al., 1998) and 915R (Stahl and Amann, 1991) and the 2011 library was generated with primers 20F-958R (Delong, 1992), resulting in 120 high quality Sanger sequences for phylogenetic reconstruction. The cloned SSU rRNA gene sequences were deposited in GenBank under accession numbers KY886013-KY886132.

To study the evolutionary relatedness between thermokarst sediment Archaea and known Archaea lineages, the SSU rRNA gene sequences were aligned (Silva's SINA aligner v 1.2.11 under the Archaea variability profile; Pruesse et al., 2012) with a reference set of archaeal rRNA genes. The reference set was built from the closest matches from GenBank, Silva (123), and cultured organisms for each OTU from this study, as well as one representative from each genus for all genera with a sequenced genome in either the NCBI or JGI-IMG databases (Hug et al., 2016). The alignment was edited as follows: columns containing ≥95% gaps were removed, and the alignment was trimmed at the 5′ and 3′ ends based on this study's sequence lengths, to a final length of 662 bp using Geneious version 8 (Kearse et al., 2012). The alignment contained a total of 267 sequences, including 77 from this study. A maximum likelihood phylogenetic tree was built using the RAxML-HPC (Stamatakis, 2014) BlackBox algorithm with default parameters for nucleotide alignments, available through the CIPRES (Cyberinfracstructure for Phylogentic Research) Science Getaway (https://www.phylo.org/). Given the short amplicon size of the archaeal iTag sequences, they were not used in tree reconstruction. However, we included SSU rRNA gene sequences of the closest neighbors (>90% identity) to the iTag sequences present in the Silva database (123) in the phylogenetic tree.

## Results

### β-diversity of the microbial assemblage among lake study sites

**Figure 1:**
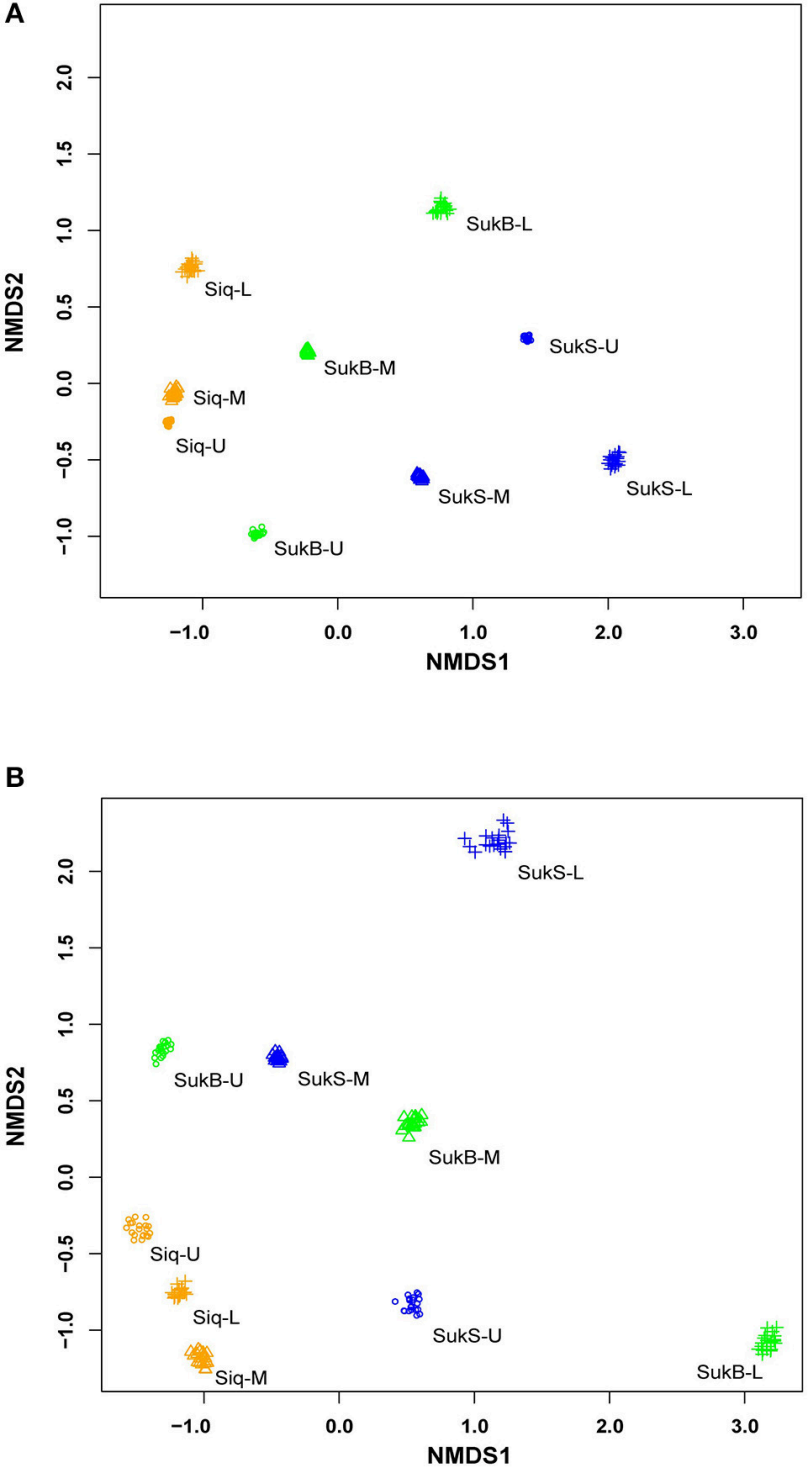
Non-metric multidimensional scaling of community dissimilarity (Bray-Curtis) calculated from **(A)** extracted DNA; stress (0.08) and **(B)** amplified RNA (aRNA) stress (0.138).

Sequences were clustered into operational taxonomic units (OTUs; distance of 0.03) by sequence type [i.e., DNA, amplified RNA (aRNA), RNA] forming an average of 1,397 ± 288 DNA OTUs, 1,397 ± 366 aRNA OTUs, and 1,447 ± 232 RNA OTUs; excluding doubletons and singletons (Table S1). Patterns of dissimilarity within and among sites (β***-***diversity) were revealed with non-metric multidimensional scaling (NMDS) ordination analysis of Bray-Curtis dissimilarity (20 *in-silico* re-samplings) of DNA and amplified RNA (aRNA) iTag occurrences from the three study sites (Figure 1). Comparisons of DNA iTags suggested distinctions of the microbial composition—such that differences were significant between Siq and SukS (Mann–Whitney test, *p* = 0.0001, significant in 1,000/1,000 resamples), compared to the difference between Siq and SukB. Significant differences were also found between SukB and SukS (Mann-Whitney test, *p* = 0.0053, significant in 1,000/1,000 resamples). The NMDS analysis based on aRNA iTag sequences (Figure 1B) showed that Siq samples clustered together, compared to SukB and SukS data sets. However, the Mann-Whitney test indicated that the aRNA sequences from the three sites were not significantly different over all *in-silico* re-samplings.

### Microbial assemblage composition of the potentially active organisms

**Figure 2:**
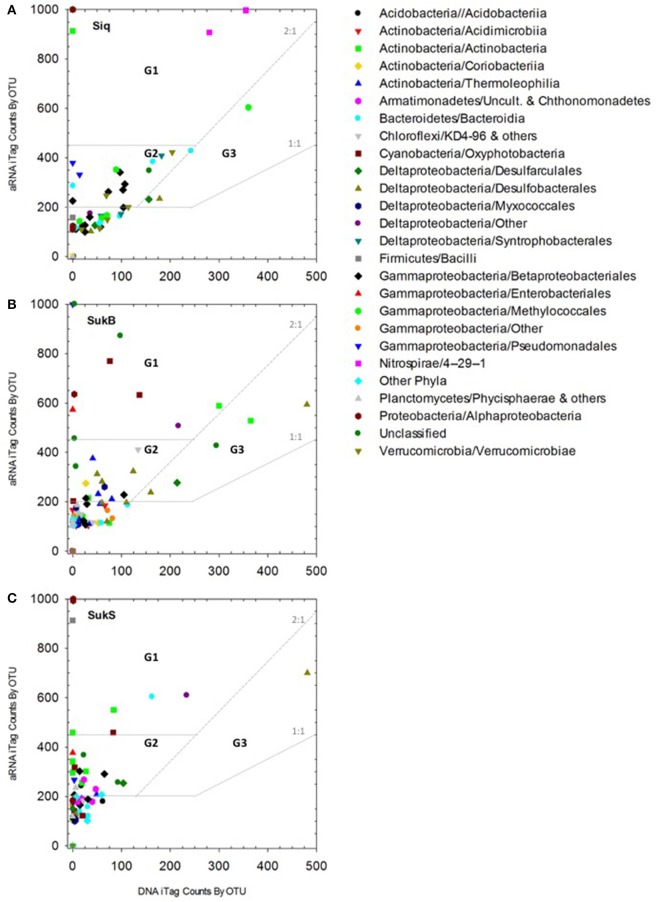
**(A–C)** aRNA resampled iTags that were significantly higher (*p* < 0.00019) than DNA resampled iTags at each of the three sites (panels: Siq, SukB, and SukS). Taxonomic affiliations to Class and/or Order are indicated by symbols in the inset legend. Three groups signaling OTUs with potentially relevant functions were identified as follows. Group 1 (G1): aRNA to DNA ratios > 2 and aRNA occurrence levels over 500. G2 OTUs: aRNA to DNA ratios > 2 and aRNA levels between 250 and 500. G3 abundant OTUs: aRNA to DNA ratios between 1 and 2 with aRNA levels ≥250 and DNA levels ≥250. OTUs whose aRNA iTags were exceptionally higher (>1,000) than their DNA counts (≤5) were set to 1,000. These corresponded to G1 OTUs including an Actinobacteria OTUs in Siq, two Alphaproteobacteria OTUs (Siq and SukS), and one Cyanobacteria OTU and one Gammaproteobacteria OTU in SukB with aRNA occurrences above 1,000 and DNA < 5. Other G1 OTUs with DNA occurrences <5 included three Actinobacteria OTUs (SukS), two Alphaproteobacteria OTUs (SukB and SukS), one Bacteroidetes OTU (Siq), two Cyanobacteria OTUs (SukB and SukS), three Gammaproteobacteria OTUs (Siq, SukB, SukS); and one Firmicutes OTU (SukS) and an Actinobacteria OTU (Siq) with aRNA occurrences ~900.

We observed a clear, positive relationship between aRNA and DNA iTag levels across the different taxa detected. The integrity of the relationship between the aRNA and RNA data sets showed best coherence for those sequences that were highly represented in the data set, while the relationship fell off at lower levels of detection (Figure S1). 7 G1 OTUs, 23 G2 OTUs, and 4 G3 OTUs with aRNA to DNA ratios >1 were identified as taxa with potentially important functions in these coring sites (Figure 2, Table 2). Eleven to sixteen potentially active OTUs and different taxonomic groups (categorized to the family level where possible) fell into G1-3 at each site, with 9 taxonomic groups identified in Siq, 8 in SukB, and 11 in SukS. A higher number of OTUs unique to a site (7) were found in Siq and SukB (6), while SukS, only had 4. The two Sukok sites shared 6 OTUs, only one of which was also active in Siq. In fact, unclassified OTUs were the only ones found to be common among the three coring sites (Siq G2, SukB G1-3, and SukS G2). The SukS data set was generally skewed toward OTUs with higher ratios, as the DNA sequence occurrence levels were low on average.

**Table 2:** Potentially active OTUs per sample and their taxonomy following Silva 132 taxonomy.

**OTU**	**Identity**	**Phylum**	**Class**	**Order**	**Family**	**Genus**	**Sample**	**Group**
**4**	**99.6**	**Nitrospirae**	**4–29–1**	**Unclassified**	**Unclassified**	**Unclassified**	**Siq-U/M**	**G1**
68	95.8	Bacteroidetes	Bacteroidia	Bacteroidales	Bacteroidetes vadinHA17	Unclassified	Siq-M/L	G2
5	98.5	Proteobacteria	Deltaproteobacteria	Syntrophobacterales	Syntrophaceae	Desulfobacca	Siq-L	G2
9	99.2	Proteobacteria	Gammaproteobacteria	Betaproteobacteriales	Burkholderiaceae	Pelomonas	Siq-U	G2
34	97.7	Proteobacteria	Gammaproteobacteria	Betaproteobacteriales	Hydrogenophilaceae	Thiobacillus	Siq-U/M	G2
71	97.3	Proteobacteria	Gammaproteobacteria	Betaproteobacteriales	Rhodocyclaceae	Thauera	Siq-U	G2
35	98.8	Proteobacteria	Gammaproteobacteria	Methylococcales	Methylomonaceae	Crenothrix	Siq-U	G2
31	99.2	Proteobacteria	Gammaproteobacteria	Pseudomonadales	Moraxellaceae	Acinetobacter	Siq-M	G2
97	86.6	Unclassified	Unclassified	Unclassified	Unclassified	Unclassified	Siq-L	G2
77	94.2	Verrucomicrobia	Verrucomicrobiae	Pedosphaerales	Pedosphaeraceae	Unclassified	Siq-L	G2
21	99.2	Proteobacteria	Gammaproteobacteria	Methylococcales	Methylomonaceae	Methylobacter	Siq-U	G3
**12**	**98.5**	**Actinobacteria**	**Actinobacteria**	**Micrococcales**	**Intrasporangiaceae**	**Tetrasphaera**	**SukB-U/M**	**G1, G3**
**119**	**99.2**	**Cyanobacteria**	**Oxyphotobacteria**	**Leptolyngbyales**	**Leptolyngbyaceae**	**Chamaesiphon PCC-7430**	**SukB-U**	**G1**
**40**	**96.9**	**Cyanobacteria**	**Oxyphotobacteria**	**Nostocales**	**Xenococcaceae**	**Pleurocapsa PCC-7319**	**SukB-U**	**G1**
**10**	**99.2**	**Proteobacteria**	**Deltaproteobacteria**	**MBNT15**	**Unclassified**	**Unclassified**	**SukB-M**	**G1**
**322**	**88.5**	**Unclassified**	**Unclassified**	**Unclassified**	**Unclassified**	**Unclassified**	**SukB-M**	**G1**
98	96.5	Actinobacteria	Coriobacteriia	OPB41	Uncultured	Uncultured	SukB-L	G2
24	97.7	Actinobacteria	Thermoleophilia	Gaiellales	Uncultured	Uncultured	SukB-M	G2
43	94.6	Chloroflexi	KD4-96	Uncultured	Uncultured	Uncultured	SukB-M	G2
7	97.7	Proteobacteria	Deltaproteobacteria	Desulfobacterales	Desulfobacteraceae	SEEP-SRB1	SukB-U	G2
237	98.8	Proteobacteria	Deltaproteobacteria	Desulfobacterales	Desulfobacteraceae	Sva0081 sediment group	SukB-U	G2
62	99.6	Proteobacteria	Deltaproteobacteria	Desulfobacterales	Desulfobulbaceae	[Desulfobacterium] catecholicum group	SukB-M	G2
222	96.5	Proteobacteria	Deltaproteobacteria	Myxococcales	MidBa8	Unclassified	SukB-L	G2
267	91.5	Unclassified	Unclassified	Unclassified	Unclassified	Unclassified	SukB-U	G2
23	97.3	Proteobacteria	Deltaproteobacteria	Desulfarculales	Desulfarculaceae	Desulfatiglans	SukB-L	G3
15	98.5	Proteobacteria	Deltaproteobacteria	Desulfobacterales	Desulfobacteraceae	Uncultured	SukB-U	G3
8	95.0	Unclassified	Unclassified	Unclassified	Unclassified	Unclassified	SukB-L	G3
**12**	**98.5**	**Actinobacteria**	**Actinobacteria**	**Micrococcales**	**Intrasporangiaceae**	**Tetrasphaera**	**SukS-M**	**G1**
**13**	**95.4**	**Bacteroidetes**	**Bacteroidia**	**Sphingobacteriales**	**Lentimicrobiaceae**	**Unclassified**	**SukS-L**	**G1**
**10**	**99.2**	**Proteobacteria**	**Deltaproteobacteria**	**MBNT15**	**Unclassified**	**Unclassified**	**SukS-L**	**G1**
79	97.7	Actinobacteria	Actinobacteria	Micrococcales	Cellulomonadaceae	Actinotalea	SukS-U	G2
65	99.2	Actinobacteria	Actinobacteria	Micrococcales	Microbacteriaceae	Cryobacterium	SukS-U	G2
187	95.0	Armatimonadetes	Uncultured	Uncultured	Uncultured	Uncultured	SukS-L	G2
40	96.9	Cyanobacteria	Oxyphotobacteria	Nostocales	Xenococcaceae	Pleurocapsa PCC-7319	SukS-M	G2
23	97.3	Proteobacteria	Deltaproteobacteria	Desulfarculales	Desulfarculaceae	Desulfatiglans	SukS-L	G2
9	99.2	Proteobacteria	Gammaproteobacteria	Betaproteobacteriales	Burkholderiaceae	Pelomonas	SukS-M	G2
34	97.7	Proteobacteria	Gammaproteobacteria	Betaproteobacteriales	Hydrogenophilaceae	Thiobacillus	SukS-U	G2
370	86.5	Unclassified	Unclassified	Unclassified	Unclassified	Unclassified	SukS-L	G2
148	89.6	Unclassified	Unclassified	Unclassified	Unclassified	Unclassified	SukS-L	G2
57	98.1	Proteobacteria	Deltaproteobacteria	Desulfobacterales	Desulfobulbaceae	Uncultured	SukS-L	G3

*Group 1 OTUs in bold*.

Of the OTUs unique to Siq, one G1 OTU was affiliated with the Nitrospirae (OTU 4 in Siq-U), while two OTUs related to Methylomonaceae fell into G2 (OTUs 35) and G3 (OTU 21) (Figure 2A). OTUs affiliated with Syntrophaceae (OTU 5) and Pedosphaeraceae (OTU 77), were also unique to the Siq G2 group. Additional G2 Siq OTUs included Hydrogenophilaceae (OTU 34) and Burkholderiaceae (OTU 9), other Gammaprotoebacteria (OTUs 31 and 71), Bacteroidetes (OTU 68), and unclassified Bacteria (OTU 97).

The SukB data set had G1 OTUs affiliated with Cyanobacteria in the families Xenococcaceae and Leptolynbyaceae (OTUs 40 and 119), Intrasporangiaceae in the Actinobacteria (OTU 12), in addition to an unclassified Deltaproteobacteria (OTU 10) and unclassified Bacteria (OTU 322) (Figure 2B). SukB G2 OTUs were associated with uncultured Actinobacteria (OTUs 24 and 98), Desulfobacteraceae (OTUs 7 and 237), Desulfobulbaceae (OTU 62), the family MidBa8 in the Myxococcales (OTU 222), and an unclassified Bacteria (OTU 267). An uncultured G2 Chloroflexi (OTU 43) affiliated with a new candidate class, was unique to this site. G3 OTUs were affiliated with Intrasporangiaceae in the Actinobacteria (OTU 12), Desulfobacteraceae (OTU 15), Desulfarculaceae (OTU 23), and unclassified Bacteria (OTU 8).

Group 1 SukS OTUs were related to Intrasporangiaceae (OTU 12), Lentimicrobiaceae (OTU 13), and unclassified Deltaproteobacteria (OTU 10) (**Figure 4C**). G2 OTUs included Actinobacteria in the families Microbacteriaceae and Cellulomonadaceae (OTUs 65 and 79), uncultured Armatimonadetes (OTU 187), Xenococcaceae (OTU 40), Desulfarculaceae (OTU 23), Burkholderiaceae (OTU 9), Hydrogenophilaceae (OTU 34), and unclassified Bacteria (OTUs 148 and 370). An OTU (57) related to Desulfobulbaceae was the sole OTU in G3.

### Thermokarst lake sediment-associated archaeal assemblage composition

**Figure 3:**
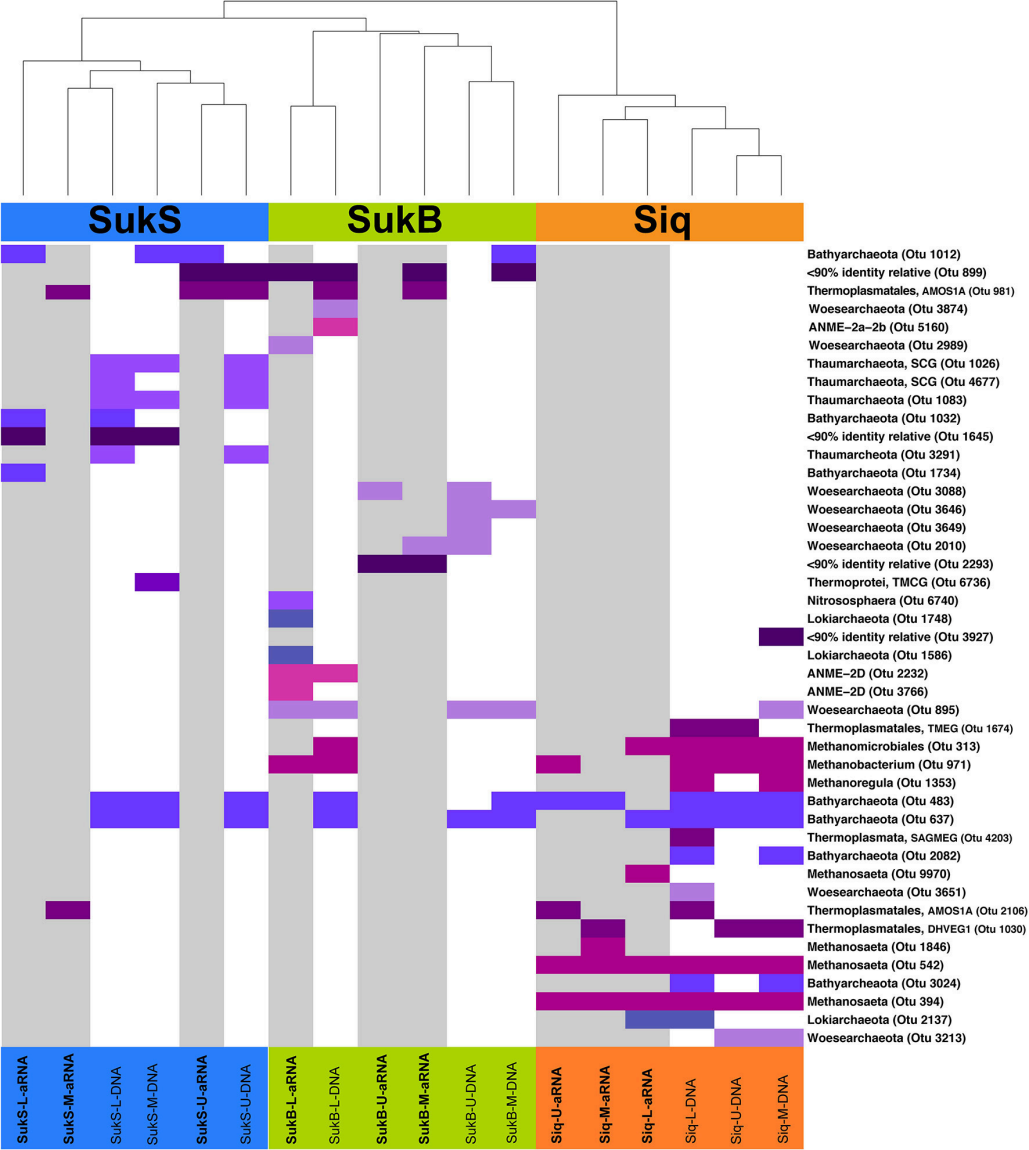
Two-dimensional hierarchical clustering (average linkage) analysis of iTag OTUs based on non-parametric Spearman's rank correlations. Abundances were rank-transformed within each resampling. Average neighbor clustering of average rank correlation was used to order OTUs and samples. Only OTUs that were observed in >95% of re-sampled datasets are shown. Sampling sites are shown in color at the top of the graph along with dendogram showing clustering relationship between the sampled aRNA or DNA where SukS samples are in blue, SukB in green, and Siq in orange. The presence of different Archaeal OTUs are shown by colored bars, with those samples represented by aRNA shown with a gray background. Different archaeal phyla are indicated by different shades of blue to purple colors. Different groups of Euryarchaeota (All methanogen-related OTUs, Thermoplasmatales, and ANME OTUs were also indicated by different colors magenta, dark purple, and pink, respectively. Taxonomic affiliation was confirmed by phylogenetic analysis of their closest neighbors (mostly > 97% identity) in the Silva Database (123).

The first indication of high levels of archaeal gene sequence diversity was revealed by PCR-DGGE of archaeal V3 SSU rRNA gene fragments from samples collected in 2010 from discrete depths of the four lakes surveyed (Table 1; Figures S2a,b). Differences between Siq10 and Suk10 lake sediments (Figure S2c) and a high number of archaeal phylotypes per site (18–43) were observed by DGGE. Subsequent analyses based on SSU rRNA gene (DNA) and amplified SSU rRNA (aRNA) iTag sequences from Siq, SukB, and SukS 2011 samples indicated that archaeal OTUs comprised a diverse, but low proportion of the iTag sequences (~1% OTUs excluding singletons and doubletons). Yet, resampling of the data set reproducibly indicated their presence and suggested a distinct composition between the sediments from all three sites. Two-dimensional hierarchical clustering (average linkage) analysis of iTag OTUs based on non-parametric Spearman's rank correlations demonstrated the patterns clearly (Figure 3). Not only were iTag sequences different between sites, but their phylogenetic affiliations were often different; only OTUs related to the Bathyarchaeota and the order Thermoplasmatales in the Euryarchaeota were present at all three sites. OTUs affiliated with Euryarchaeota methanogens were dominant among the DNA and aRNA sequences at the Siq coring site, while OTUs affiliated with Woesearchaeota were only found in DNA and aRNA sequences at the SukB coring site.

Noteworthy distinctions among sites included OTUs affiliated with *Methanobacterium, Methanosarcina, Methanosaeta*, and Methanocellales in Siq, while SukB only had a single methanogen-OTU and they were absent in the SukS data set. The SukB iTag composition differed substantially from the other sites: OTUs related to anaerobic methanotrophs of the ANME-2d group and other Euryarchaeota in the order Thermoplasmatales (AMOS1A-4113-D04) were observed, in addition to representatives of the Lokiarchaeota, Woesearchaeota, Thaumarchaeota and Bathyarchaeota. At SukS, Bathyarchaeota-, Thermoplasmatales-, and Thaumarchaeota-related OTUs were present. Additionally, three archaeal OTUs without close relatives (<90% identical in the Silva database) were present in SukB and SukS.

### Phylogeny of arctic archaea inferred from the SSU rRNA gene

**Figure 4:**
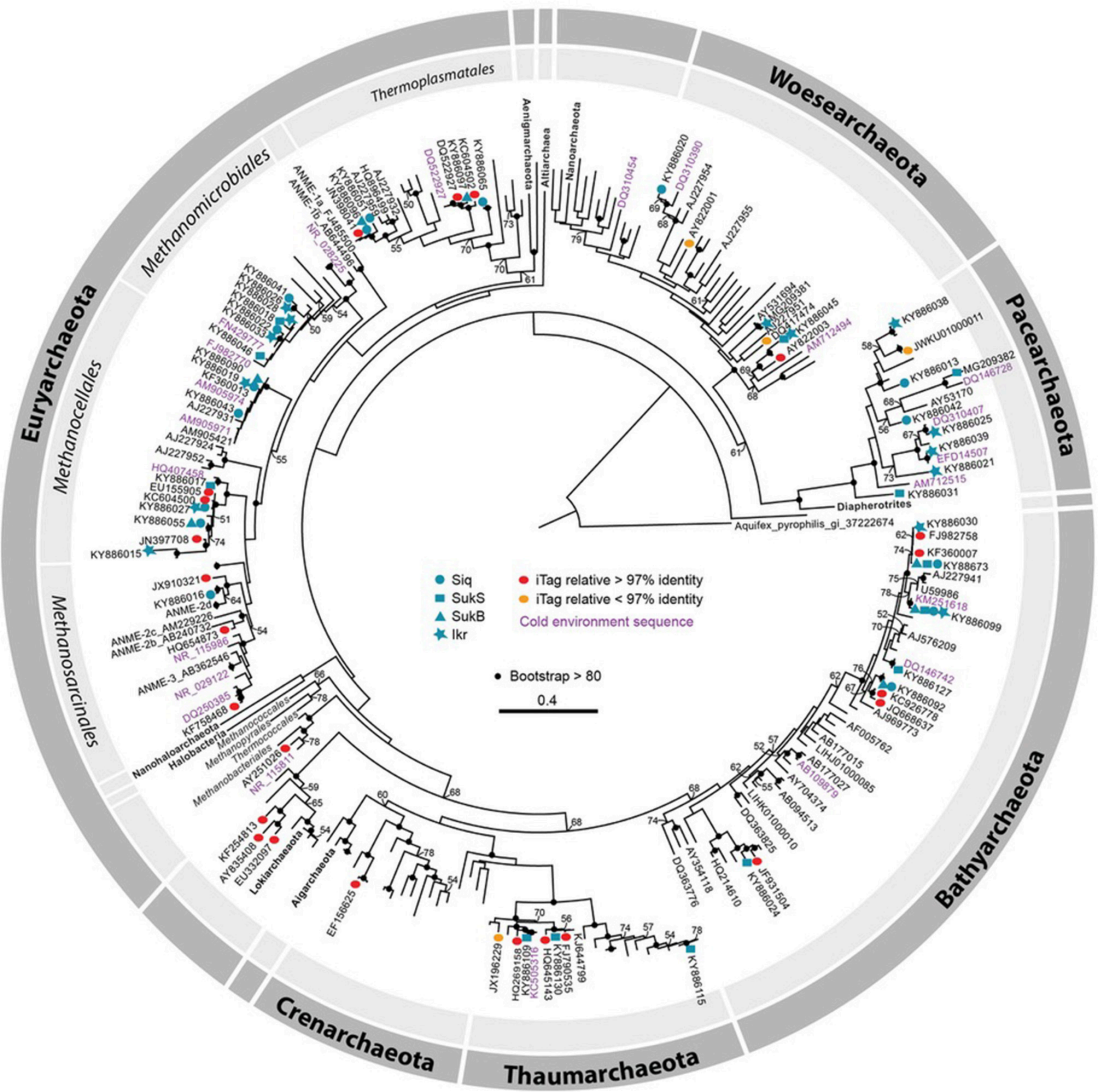
Maximum likelihood phylogenetic tree of SSU rRNA gene (626–660 bp). Clone library sequences from both years (2010 and 2011) are coded by symbol as follows: Siq—circles, SukB—hexagons, SukS—squares, and Ikroavik (Ikr)—stars, followed by their corresponding accession numbers. Note that only one representative sequence was included for each taxon in the phylogenetic tree, and all the other sequences are available (KY886013–KY886132). Silva database sequences with ≥97% identity to iTag sequences are shown as red ovals and sequences with ≤97% identity are shown as orange ovals. Environmental sequences or culture isolates in the GenBank that were ≥97% similar to Sanger sequences, and that were obtained from cold environments (e.g., Canadian Arctic, Laptev Sea, Alaska, Antarctica, and others) are represented in pink and nearest neighboring sequences obtained in GenBank or the SILVA database are in purple. Only bootstrap values ≥50 are shown, and bootstrap values ≥80% are represented by black circles. For a full description of the SSU rRNA gene sequences on the tree, including sequences from the JGI IMG database, see Figure S4.

Phylogenetic analysis of archaeal SSU rRNA gene sequence region of overlap between the two primer sets (~662 bp) indicated that thermokarst lake sediment Archaea from 2010 and 2011 samples span the a wide range of Archaea phyla recognized to date (Figure 4). Only a few iTag-related sequences fell into clusters not covered by the clone sequences (e.g., Methanobacteriales in the Euryarchaeota, Lokiarchaeota, and Crenarchaeota). The majority of arctic thermokarst lake Archaea cloned sequences and iTag sequences were related to members of the phylum Bathyarchaeota (Meng et al., 2014; Evans et al., 2015), particularly those originating from Sukok Lake. Less well represented were iTag-related sequences within the Thaumarchaeota, a sister group to the Bathyarchaeota*;* and only one iTag-related sequence was affiliated with Crenarchaeota. Methanogen representatives, Methanosarcinales, Methanomicrobiales, Methanocellales, and a new order of methanogens related to the Thermoplasmatales (Borrel et al., 2013) were found among cloned and iTag-related sequences. Several Thermoplasmatales-related sequences in the Euryarchaeota were also observed in cloned and iTag sequences from Siq and SukB. Only one cloned sequence was related to anaerobic methanotrophs of the ANME-2d group, though several iTag-related sequences grouped in this part of the tree too. Sequences detected in both clone and iTag libraries affiliated with the Woesearchaeota and the Pacearchaeota clustered with numerous sequences from other arctic environments (i.e., Canadian artic lakes and rivers, and the Beaufort Sea). Sequences assigned to the candidate phylum Lokiarchaeota, which has been suggested to hold common ancestry with the Eukaryotes (Spang et al., 2015; Mariotti et al., 2016), were also detected.

## Discussion

We set out to study the microbial assemblages that may be actively involved in geochemical processes relevant to thermokarst lake sediments. Siq has been reported to have a biogenic CH_4_ signature and higher levels of TOC and iron (Fe) compared to Sukok, which has a natural gas seep near SukS with a thermogenic CH_4_ signature, and higher levels of nitrate (${\text{NO}}_{3}^{-}$) and ${\text{SO}}_{4}^{2-}$ in pore waters at both sites (Matheus Carnevali et al., 2015). Between the two sites in Sukok, the geochemical differences appeared to be modest, though low levels of biogenic CH_4_ production potential was demonstrated at SukB in addition to higher Fe levels compared with SukS (Matheus Carnevali et al., 2015). Based on these data, and the remarkable presence of biogenic CH_4_ in an area with natural gas reserves (thermogenic CH_4_), we focused our attention on these contrasting coring sites. Thus, we hypothesized that the microbial assemblage composition, the potentially active fraction (indicated by high aRNA:DNA levels) in particular, differed following geochemistry.

### Microbial assemblage structure and composition

NMDS patterns based on DNA and aRNA suggested differences in microbial assemblage structure among the three sites that were consistent with geochemical profiling of sediment cores (Matheus Carnevali et al., 2015). The lack of statistically significant differences between lakes in the analysis of aRNA sequences may be explained by a high variation in assemblage structure between depths and sites at Sukok. Another potentially confounding influence enhancing distance between sediment samples could be inactive organisms reflected by the DNA-based iTag surveys (Juottonen et al., 2008), though the aRNA results here were largely in support of differences between coring sites.

At the Phylum or Class levels Siqlukaq and Sukok thermokarst lake microbial assemblages are representative of other arctic permafrost and peatland environments. Actinobacteria, Bacteroidetes, and Proteobacteria, which dominated here, also tend to dominate other permafrost environments (Yergeau et al., 2010; Frank-Fahle et al., 2014; Johnston et al., 2016), although Deltaproteobacteria and not Alphaproteobacteria or Betaproteobacteria were more abundant in this study. Nitrospirae were found to be abundant in different kinds of Northern wetlands (Wilhelm et al., 2011; Urbanová and Bárta, 2014). Other studies have also reported Chloroflexi and Cyanobacteria (Ganzert et al., 2014; Hultman et al., 2015). Verrucomicrobia were also found in a Northern wetland, where Armatimonadetes were considered “rare” members of the community (Serkebaeva et al., 2013). Bacteria and Archaea affiliated with the same phyla observed in this study have also been found in Russian permafrost (Shcherbakova et al., 2016), freshwater lakes of the Yunnan Plateau (Zhang et al., 2015), and most importantly in Toolik Lake on the North Slope of Alaska (Crump et al., 2012). Also, Acidobacteria were less abundant in these mineral lake sediments than in acidic peatlands (Wilhelm et al., 2011; Tveit et al., 2012; Serkebaeva et al., 2013).

### Major inferences of potentially active bacteria and putative functions in lake sediments

The value of using both rRNA and rRNA coding DNA sequence information in this study increased confidence in the interpretation of the molecular ecological results. We observed correlations between RNA and DNA frequencies among different taxa (Campbell et al., 2011) and used rRNA: DNA ratios >1 as a proxy for activity following Wilhelm et al. (2014) and Hultman et al. (2015). These OTUs (Groups 1–3) were considered as candidates for potentially contributing important ecosystem functions and were identified as those with some form of activity. That is, microorganisms may increase the number of ribosomes not only in preparation for protein synthesis to meet real-time growth and production needs, but also as an adaptive strategy to cope with rapid shifts in environmental conditions, or before entering dormancy (Blazewicz et al., 2013). However, we expect that it is more likely to overestimate false negatives (i.e., classify organisms as dormant) than false positives (i.e., classify organisms as active) in communities comprising populations with variable growth strategies (Steven et al., 2017).

Diverse bacterial assemblages with taxa that may be actively involved in C, S, and N cycling were found at all three sediment coring sites. Differences in biogeochemical functional potential between the sites were revealed using rRNA-derived sequence analyses. Group 1 OTUs demonstrated this pattern, where N- or S-cycling Nitrospirae in Siqlukaq; photosynthetic, N_2_-fixing Cyanobacteria in SukB; organic matter decomposing Actinobacteria and Bacteroidetes in SukS; as well as Actinobacteria and unclassified Deltaproteobacteria (MBNT15) in both Sukok sites, suggested different processes at each site.

Functional inferences regarding methane oxidation in particular, appear to be unique to Siq. Here, the data suggest the prevalence (Figures S2a, S3b) of G2 and G3 OTUs affiliated with *Methylobacter* and *Crenothrix*. Some *Crenothrix* can be responsible for the oxidation of dissolved iron (Fe^2+^) (Emerson et al., 2010; Hedrich et al., 2011) while others are also capable of aerobic and anaerobic CH_4_ oxidation (Stoecker et al., 2006; Oswald et al., 2017). When these sequences were compared to the nr database at NCBI using BLAST, these two OTUs (21 and 71) matched *Methylobacter tundripaludum* (95–100% similarity, 98–100% coverage). The discrepancy in taxonomic affiliations are currently unresolved—regardless, both *Crenothrix* and *Methylobacter* are type I methanotrophs. *M. tundripaludum* is a psychrophile isolated from High Arctic wetland soil (Wartiainen et al., 2006; Svenning et al., 2011) and it has also been observed in Lake Qalluuraq, 60 miles to the South of Siq (one of our initial lakes surveyed for archaeal diversity; He et al., 2012a). The potential metabolisms of these microorganisms reflect the geochemical resources in Siq sediments, which contain the highest amounts of Fe^2+^ and CH_4_ of the three sites (Matheus Carnevali et al., 2015).

In a similar vein, though less clear of the functional role(s), Siq contained G1 Nitrospirae affiliated OTUs, not closely related to cultivated members of this group. Nitrospirae are metabolically versatile bacteria that include chemoautotrophic nitrite (${\text{NO}}_{2}^{-}$) oxidizers (Lücker et al., 2010), complete ammonia (NH_3_) oxidizers (Daims et al., 2015; Koch et al., 2015), and S- or ${\text{SO}}_{4}^{2-}$-reducing bacteria (Sekiguchi et al., 2002, 2008).

OTUs that could participate in complex organic matter degradation processes at the coring sites included Actinobacteria and Bacteroidetes in addition to other less well represented, yet potentially active taxa. Bacteroidetes OTUs appeared to be active in SukS (G1) and Siq (G2), and are known to be associated with aerobic and anaerobic cellulose, chitin, and pectin degradation (Kirchman et al., 2014). G1-G3 Actinobacteria OTUs in SukS and SukB (Table 2) may also be contributing to complex C compound degradation (Barka et al., 2016). These sequence-based observations are consistent with analysis of organic matter composition in Suk, which indicated that in comparison to Siq, there was a greater contribution of free hydrocarbons derived from terrestrial plants in these sediments (Matheus Carnevali et al., 2015). At Siq, Verrucomicrobia G2 OTUs were also unique and could contribute to organic matter decomposition with saccharolytic metabolic potential (Bergmann et al., 2011; Hugenholtz et al., 2016).

Sulfur-cycling Deltaproteobacteria in Suk were affiliated with dissimilatory ${\text{SO}}_{4}^{2-}$ reducers, while Siq Deltaproteobacteria were affiliated with organisms that can be found in syntrophy with methanogens or other H_2_-consuming bacteria. These organisms could carry out subsequent steps in organic matter mineralization (e.g., fermentation and anaerobic respiration) in the lake sediments. The potential for reducing ${\text{SO}}_{4}^{2-}$, sulfite (${\text{SO}}_{3}^{2-}$), and thiosulfate to hydrogen sulfide (H_2_S) could be inferred from the presence of groups known to perform these functions, with high aRNA to DNA ratios at both Sukok coring sites. Conversely, at the Siq coring site, Syntrophaceae (G2 OTUs) and Hydrogenophilaceae (G2 OTUs) were the OTUs that could potentially be attributed to S cycling. The Hydrogenophilaceae OTU was affiliated with *Thiobacillus*, chemoautotrophic bacteria capable of coupling Fe or S oxidation with denitrification (Beller et al., 2006).

### Expansive diversity of archaea and their potential roles in arctic lake sediments

We investigated the previously unknown structure and phylogeny of Archaea in sediments of four arctic thermokarst lakes, and uncovered high archaeal diversity in these sediments. These findings emphasize the 2011 coring sites Siq, SukS, and SukB, even though Ikr10-O was also included in the phylogenetic analyses. Based on SSU rRNA gene clone library sequencing and SSU rRNA gene and SSU rRNA iTag sequencing, distinct Archaea compositions were observed at the three coring sites. These differences were also reflected in the potentially active Archaea (Figure 3). Overall, Archaeal OTUs comprised a low proportion of the total iTag sequences surveyed, which is comparable to other arctic soil and permafrost environments (Wilhelm et al., 2011; Tveit et al., 2012). Likewise, we found higher diversity and higher numbers of potentially active methanogen OTUs in sediments with higher organic levels, similar to findings in the water column of anoxic sub-arctic thaw ponds in Quebec, Canada (Crevecoeur et al., 2016).

OTUs affiliated with methanogens dominated at the Siq coring site, while OTUs affiliated with other archaeal groups were distributed in the Suk sediments. This partition of methanogens mirrored the potential for biological CH_4_ production observed in Siq and SukB sediments (Matheus Carnevali et al., 2015). The phylogenetic diversity of methanogens in Siq also suggested the potential for multiple pathways of methanogenesis. For example, methanogens in the orders Methanosarcinales and Methanomicrobiales are capable of acetoclastic or hydrogenotrophic methanogenesis, while members in the Methanocellales only produce CH_4_ from H_2_ and CO_2_. Methanosarcinales and Methanomicrobiales were not only found to be potentially active here, but also in a recent study in the anoxic water column of subarctic thaw ponds in Quebec (Crevecoeur et al., 2016). Methanogens related to *Methanomassiliicoccus luminyensis* are characterized by methylotrophic methanogenesis (Dridi et al., 2012), similar to Archaea in the phylum Bathyarchaeota, which are the first group identified outside the phylum Euryarchaeota that have the potential for CH_4_ production. The Bathyarchaeota, originally the Miscellaneous Crenarchaeal Group (MCG), is a diverse (76–87.7% intragroup similarity of the SSU rRNA gene) and ubiquitous group of Archaea (Kubo et al., 2012; Meng et al., 2014) that is metabolically versatile, and includes members capable of acetogenesis (He et al., 2016; Lazar et al., 2016).

Of all three sites, SukB had the greatest diversity of archaeal phyla and SukS the lowest. OTUs in SukB were affiliated with Euryarchaeota (e.g., ANME-2d), Thaumarchaeota (e.g., *Candidatus Nitrososphaera*), Woesearchaeota, and Lokiarchaeota, while OTUs in SukS were limited to Bathyarchaeota, Thaumarchaeota, and Thermoplasmatales (AMOS1A). The presence of OTUs affiliated with ANME-2d in SukB suggested the potential for anaerobic CH_4_ oxidation in the sediments, as observed in organisms (*Candidatus Methanoperedens nitroreducens*) that belong to this group (Haroon et al., 2013), which are able to couple the reduction of Fe^3+^ and Mn^4+^ to CH_4_ oxidation (Ettwig et al., 2016). Moreover, the presence of an OTU affiliated with *Candidatus Nitrososphaera* in SukB and other Thaumarchaeota in SukS, suggested that ammonium-driven microbial processes may occur (Spang et al., 2012; Zhalnina et al., 2014). These findings, in addition to the abundance of N_2_-fixing Cyanobacteria in Sukok allow us to infer that N cycling may be active in arctic thermokarst lake sediments.

Archaeal phylogenetic analysis also revealed extensive diversity across several poorly characterized and recently recognized archaeal phyla. Here, we found sequences affiliated with recently proposed phyla Woesearchaeota and Pacearchaeota (Baker et al., 2010; Rinke et al., 2013; Castelle et al., 2015), that are currently recognized to be dominant in environments such as oligotrophic alpine Pyrenean lakes (Ortiz-Alvarez and Casamayor, 2016). Analysis of a reconstructed, almost complete Woesearchaeota genome suggested that these organisms have a saccharolytic or fermentative life style, and the ability to metabolize hydrogen (Castelle et al., 2015). Additionally, some Pacearchaeota may have the ability to fix C (Castelle et al., 2015). Interestingly, these phyla may be common to high latitudes, as they were previously recognized as (i) the Rice Cluster V (RC-V), many of which have been reported from Arctic habitats (Großkopf et al., 1998; Galand et al., 2006, 2008; Høj et al., 2008) and (ii) a second geographically widespread cluster reported from an arctic river, a stamukhi lake, and the Arctic Ocean (Galand et al., 2006, 2008), in addition to lake sediments in Germany (Glissman et al., 2004). This group was also previously regarded as Candidate division WCHD3-30 (Galand et al., 2006; Høj et al., 2008).

In summary, our work revealed distinctive bacterial assemblages in the three sites studied, which are also implicated in different biogeochemical processes. Moreover, the high diversity of Archaea observed argues for further study to establish their roles in CH_4_ production and consumption, not to mention currently unrecognized roles of some pervasive new phyla now recognized in these sediments. Arctic thermokarst lakes are sensitive to climate warming (Osterkamp, 2005; McGuire et al., 2009; Thienpont et al., 2013), and we predict that deepening of the lake talik and shifts in geomorphology might influence these lake ecosystems according to their geochemical properties. Understanding the geochemistry is only part of the picture that underlies the differences in these lakes. To fully understand how small shifts in temperature, seasonal duration, and snowfall, may cascade into shifts in ecosystem function, the regulation of microbial activities is key to predict greenhouse gas emissions.

## Author contributions

PM, CH, KH, JP, and AM contributed to the design of the study, analysis of the data, drafting of the manuscript, and approval of the content. All agree to be accountable for all aspects of the work.

### Conflict of interest statement

The authors declare that the research was conducted in the absence of any commercial or financial relationships that could be construed as a potential conflict of interest.
