# Supplementary material for: Distinct Microbial Assemblage Structure and Archaeal Diversity in Sediments of Arctic Thermokarst Lakes Differing in Methane Sources
Source: Front Microbiol. 2018 Jun 7;9:1192. doi: 10.3389/fmicb.2018.01192 (PMC6000721; doi:10.3389/fmicb.2018.01192)
Supplement: Supplementary file 1 [file Presentation_1.pdf]

Supplementary information

Methods

Nucleic acid extraction for iTag sequencing of the SSU rRNA of Bacteria and Archaea

Two grams of sediment (in duplicate) were bead beat with MoBio's proprietary solutions and phenol:chloroform:isoamyl alcohol (pH 6.5-7.0) by vortexing at maximum speed for 15 min. The organic and the aqueous phases were separated by centrifugation at 1,040 x g for 24 min, followed by a secondary precipitation step at 4 °C for 10 min to further purify the aqueous phase, and another centrifugation step under the same conditions. The resulting supernatant was split in 1.7 ml Eppendorf tubes and the extracted nucleic acids were precipitated in isopropanol at room temperature for 30 min, followed by cold centrifugation at 13,000 x g for 15 min, and a clean-up step. This last step involved 70 % ethanol at room temperature, cold centrifugation at 13,000 x g for 15 min, air-drying of the pellets (or using a Speed Vac Vacuum Concentrator at medium temperature), and final suspension of the total nucleic acids in 45-50 µL of nuclease-free water (Life Technologies, Carlsbad, CA, USA). All reagents, disposable supplies and instruments used for this protocol were RNAse-free or treated with RNaseZap decontamination solution (Life Technologies, Carlsbad, CA, USA).

Archaeal PCR and denaturing gradient gel electrophoresis analysis of 2010 samples

In order to get a rapid snapshot of the archaeal assemblage diversity and structure, primers targeting the archaeal SSU rRNA gene: 347F (this study) with GC clamp from Murray et al. (1996):

5'-

CGCCCGCCGCGCCCCGCGCCCGTCCCGCCGCCCCCGCTCCGGGCGCAGCAGGCGMG

AA -3' and UNIV519R (Muyzer et al., 1993) were used to amplify the variable region 3 (V3) with the following reaction mix: 1X Standard Taq Buffer (New England BioLabs), 2 mM $MgCl_2$, 0.2 mM of each dNTP (Qiagen), 0.5 μM of each primer, and 1.25 U Taq DNA polymerase (New England BioLabs) in a final volume of 50 μL. Thermocycler conditions were: 94 °C for 5 min, followed by 10 cycles of denaturation at 94 °C for 45 sec, touch-down primer annealing with the temperature decreasing from 65 °C to 55 °C (1 °C per cycle) for 30 sec, extension at 72 °C for 30 sec; 18 cycles of denaturation at 94 °C for 30 sec, primer annealing at 55 °C for 30 sec, extension at 72 °C for 30 sec, and a final extension step at 72 °C for 10 min. Amplification products were precipitated overnight at -20 °C with two volumes of absolute ethanol and 1/10 of the volume of 3 M sodium acetate pH 5. After centrifugation in cold for 30 min, the DNA was washed with 70% ethanol, centrifuged for 10-15 min, air-dried, or dried in a DNA speed vac (Savant), and suspended in 18 μl of sterile DI water. Two to seven PCR reaction products were pooled to reach 600 ng of DNA per sample. A 30 to 65 % gradient of denaturants (7 M urea and 40% deionized formamide in 100% concentration of denaturants) was used. Electrophoresis was run at 60 °C for 16 hrs at 62 V (Murray et al., 1996). Analyses of the banding patterns resulting from DGGE were done with the GelCompar II software (Applied Maths).

Clone libraries construction and analyses

To amplify the SSU rRNA gene of Archaea with primers 109F-915R for clone library construction we used the following PCR conditions: 1X Standard Taq Buffer, 2 mM MgCl2, 0.2 mM of each dNTP, 0.5 μM of each primer, and 1U Taq DNA polymerase in a final volume of 20 μL. Thermocycler conditions were: 94 °C for 5 min, followed by 28 cycles of denaturation at 94 °C for 1 min, primer annealing at 52 °C for 1 min, extension at 72 °C for 1 min 30 sec, and a final extension step at 72 °C for 6 min. For amplification with 20F-958R Archaea primers the

PCR reaction mix included: 1X Ampli Taq Buffer II (Life Technologies), 2.5 mM MgCl2 (Life Technologies), 0.2 mM of each dNTP, 0.5 μM of each primer, and 1.25 U AmpliTaq DNA polymerase (Life Technologies) in a final volume of 25 μL. Thermocycler conditions were: 94 °C for 10 min, followed by 35 cycles of denaturation at 94 °C for 1 min, primer annealing at 55 °C for 1 min, extension at 72 °C for 1 min, and a final extension step at 72 °C for 7 min.

The libraries were prepared with agarose gel-purified PCR amplicons using the TOPO TA cloning kit (Life Technologies), and plasmids were extracted with the Plasmid Miniprep$_{96}$ Kit (2010 library; EMD Millipore) or Qiagen 3000 BioRobot and a R.E.A.L prep (2011 library, Qiagen). The 2010 library was screened using EcoRI restriction enzyme digests. Plasmids were unidirectionally sequenced with ABI BigDye Terminator Cycle Sequencing Ready Reaction Kit V3.1 using vector primer T7 on an ABI3730 DNA Analyzer (Life Technologies). Sequences were trimmed with BioEdit v. 7.2.5 and chimeras checked with Decipher (Wright et al. 2012).

Table S1. iTag Sequence statistics. Data presented here was not normalized to correct for iTag pool sizes. Sediment intervals correspond to three sections in the first 30 cm of sediment: upper (U), middle (M), and lower (L).

Lake	Sediment Interval	Number of filtered sequences (1,213,686)			Number of OTUs at 0.03 distance (singletons and doubletons removed)			Number of singleton or doubleton OTUs at 0.03 distance		
		**DNA**	**aRNA**	**RNA**	**DNA**	**aRNA**	**RNA**	**DNA**	**aRNA**	**RNA**
**Siq**	**U**	66,997	**46,989**	51,281	1,669	**1,681**	1,645	4,152	**3,406**	3,954
	**M**	71,199	**42,585**	48,254	1,766	**1,346**	1,463	3,997	**2,260**	3,064
	**L**	67,282	**47,140**	-------	1,443	**1,685**	-------	3,102	**2,961**	-------
**SukB**	**U**	63,393	**46,861**	47,983	1,564	**1,251**	1,341	3,204	**2,324**	2,767
	**M**	63,461	**48,210**	29,397	1,449	**1,279**	1,111	3,061	**2,056**	1,951
	**L**	66,139	**41,519**	46,050	1,163	**934**	1,673	2,305	**1,072**	1,509
**SukS**	**U**	58,683	**47,850**	-------	1,099	**1,440**	-------	2,156	**1,996**	-------
	**M**	48,521	**50,672**	-------	1,533	**2,045**	-------	2,805	**3,634**	-------
	**L**	54,999	**58,221**	-------	890	**916**	-------	1,566	**2,198**	-------

Figure Legends

Figure S1. RNA versus amplified RNA (aRNA) counts from samples where both kinds of sequences were available (Siq-U/M and SukB-U/M/L), indicating a high correlation between the RNA and the amplified RNA. The integrity of the relationship between the aRNA and RNA data sets showed best coherence for those sequences that were highly represented in the data set (*i.e.,* OTUs with 100 sequences or more— abundant OTUs were closer to the one to one line), while the relationship falls off at lower levels of detection.

Figure S2. Denaturing gradient gel electrophoresis (DGGE) of the variable region 3 (V3) in the SSU rRNA gene of Archaea from April 2010 samples (A) and, October 2010 samples (O). This gel picture shows discrete sediment intervals: (a) Qalluuraq Lake (Qal10), Ikroavik (Ikr10-A or Ikr10-O) and Siqlukaq (Siq10); (b) Sukok Center (SukC10) and Sukok Seep (SukS10). Samples from Qal10, Ikr10-A, and SukC10 were not included in other analyses in this study. Genomic DNA was amplified with the Archaea-specific primer GC_347F and the UNIV519R. Most phylotypes were grouped by depth and at least one phylotype was common to all lakes. (c) UPGMA hierarchical clustering analysis of archaeal V3 region of the SSU rRNA gene profiles based on Euclidean distances. Samples for this analysis were collected in 2010. Numbers next to the lake initials are sediment intervals in cm.

Figure S3. Amplified RNA (aRNA) versus DNA counts of (a) Actinobacteria OTUs and (b) Methylococcales OTUs. OTUs with > 99 aRNA counts and > 5 DNA counts were colored according to the site (a) Siq, (b) SukB, and (c) SukS. OTUs deriving from different depths within a site were not differentiated in these plots.

Figure S4. Full phylogenetic tree (pdf).

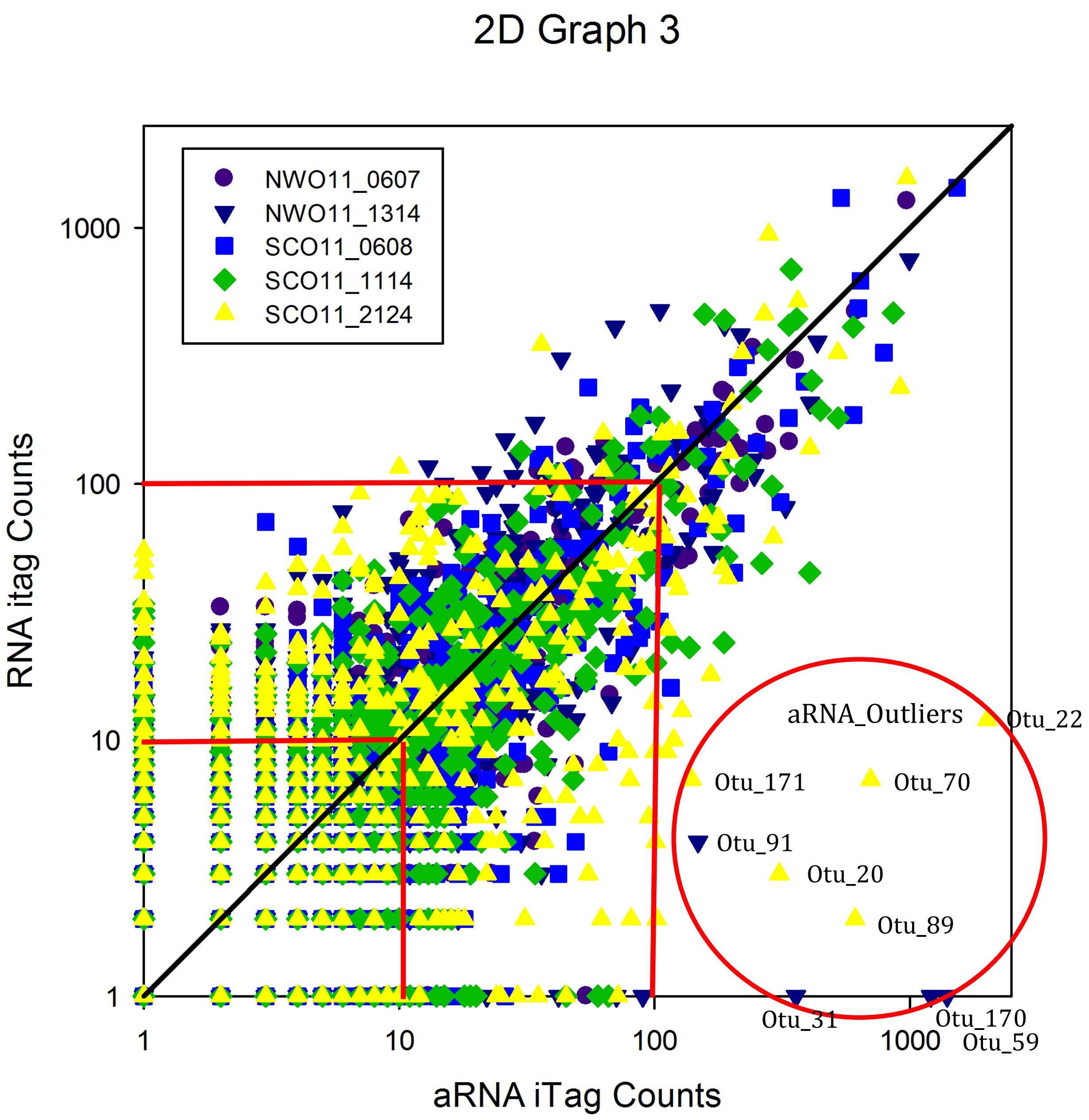
2D Graph 3
NWO11_0607
NWO11_1314
SCO11_0608
SCO11_1114
SCO11_2124
RNA itag Counts
aRNA iTag Counts
1000
100
10
1
1
10
100
1000
aRNA_Outliers
Otu_22
Otu_171
Otu_70
Otu_91
Otu_20
Otu_89
Otu_31
Otu_170
Otu_59

Figure S1

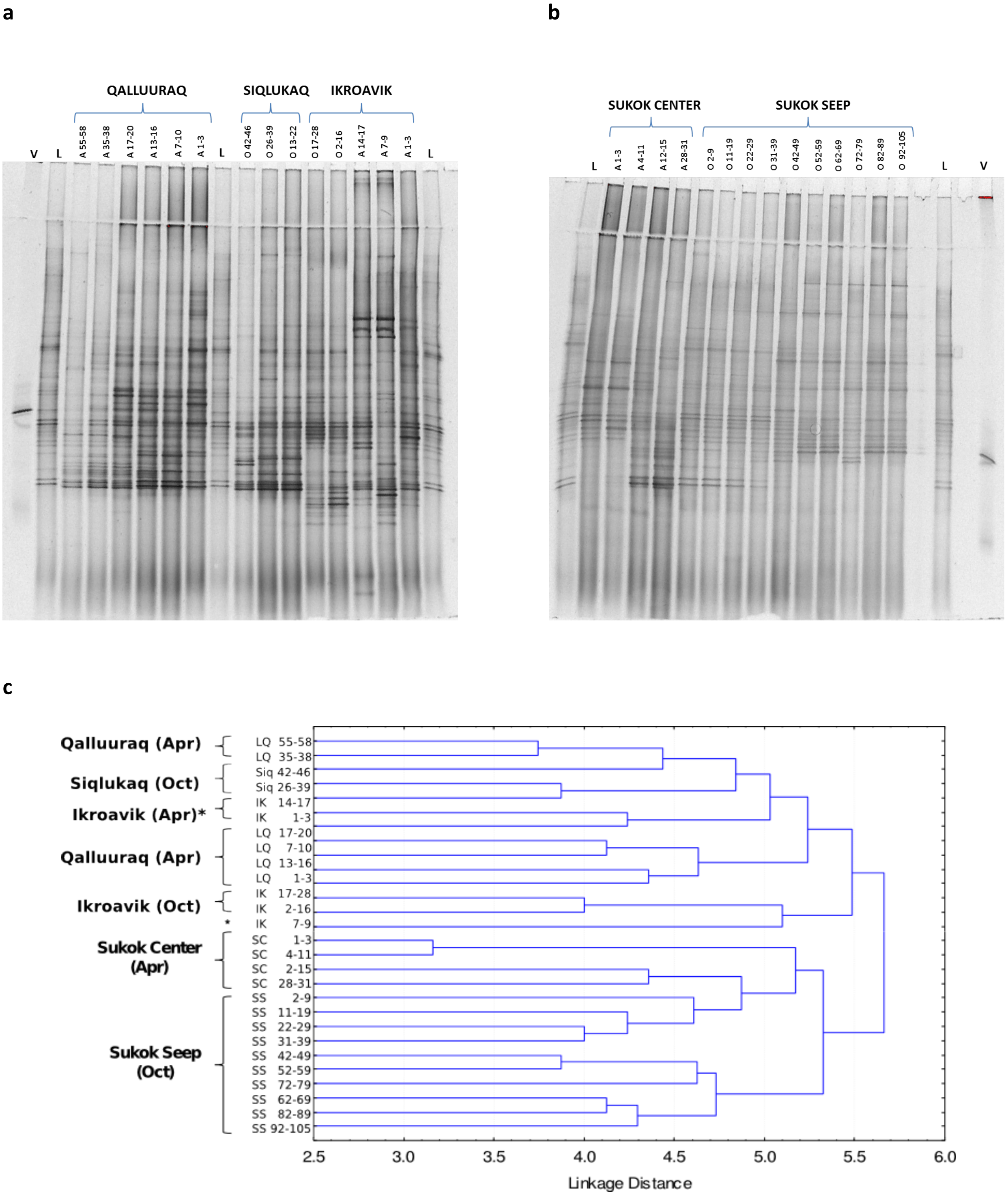
a
QALLUURAQ
SIQLUKAQ
IKROAVIK
V
L
A 55-58
A 35-38
A 17-20
A 13-16
A 7-10
A 1-3
L
O 42-46
O 26-39
O 13-22
O 17-28
O 2-16
A 14-17
A 7-9
A 1-3
L
b
SUKOK CENTER
SUKOK SEEP
L
A 1-3
A 4-11
A 12-15
A 28-31
O 2-9
O 11-19
O 22-29
O 31-39
O 42-49
O 52-59
O 62-69
O 72-79
O 82-89
O 92-105
L
V
c
Qalluuraq (Apr)
Siqlukaq (Oct)
Ikroavik (Apr)*
Qalluuraq (Apr)
Ikroavik (Oct)
*
Sukok Center (Apr)
Sukok Seep (Oct)
LQ 55-58
LQ 35-38
Siq 42-46
Siq 26-39
IK 14-17
IK 1-3
LQ 17-20
LQ 7-10
LQ 13-16
LQ 1-3
IK 17-28
IK 2-16
IK 7-9
SC 1-3
SC 4-11
SC 2-15
SC 28-31
SS 2-9
SS 11-19
SS 22-29
SS 31-39
SS 42-49
SS 52-59
SS 72-79
SS 62-69
SS 82-89
SS 92-105
2.5
3.0
3.5
4.0
4.5
5.0
5.5
6.0
Linkage Distance

Figure S2.

a

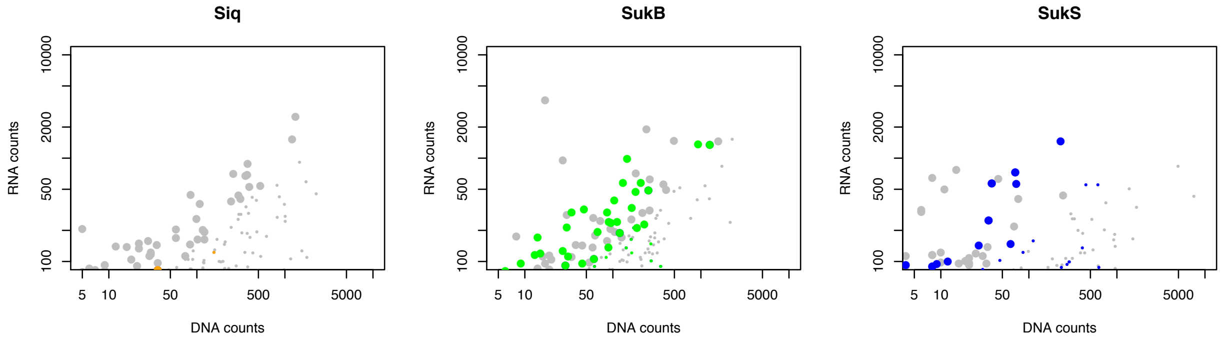
Siq
SukB
SukS
RNA counts
10000
2000
500
100
DNA counts
5
10
50
500
5000

b

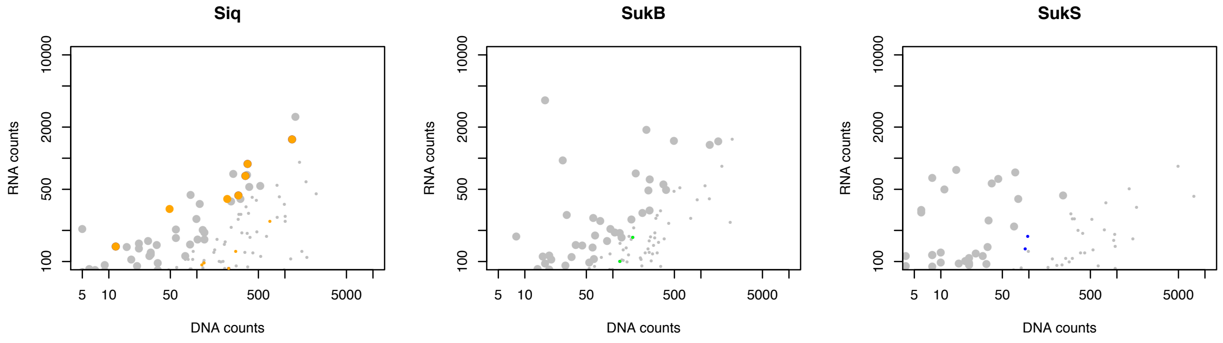
Siq
SukB
SukS
RNA counts
10000
2000
500
100
DNA counts
5
10
50
500
5000

Figure S3.

References

Murray, A.E., Hollibaugh, J.T., and Orrego, C. (1996) Phylogenetic compositions of bacterioplankton from two California estuaries compared by denaturing gradient gel electrophoresis of 16S rDNA fragments. *Appl Environ Microbiol* **62**: 2676-2680.

Muyzer, G., de Waal, E.C., and Uitterlinden, A.G. (1993) Profiling of complex microbial populations by denaturing gradient gel electrophoresis analysis of polymerase chain reaction-amplified genes coding for 16S rRNA. *Appl Environ Microbiol* **59**: 695-700.
