# Supplementary figures and images for: Distinct Microbial Assemblage Structure and Archaeal Diversity in Sediments of Arctic Thermokarst Lakes Differing in Methane Sources
Source: Front Microbiol. 2018 Jun 7;9:1192. doi: 10.3389/fmicb.2018.01192 (PMC6000721; doi:10.3389/fmicb.2018.01192)

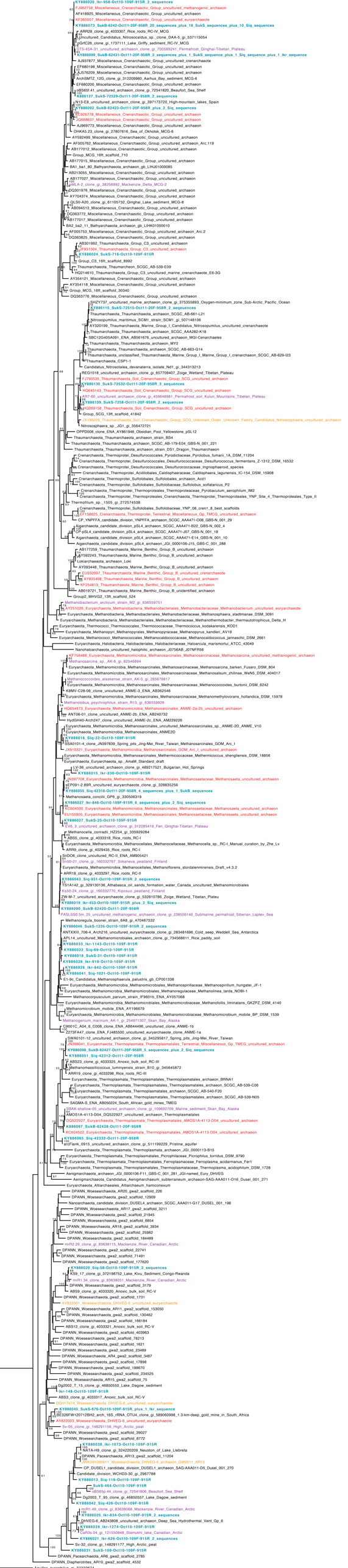

Supplement: Supplementary file 2 [file Image_1.pdf]
